# Perspective Technologies of Vaccination: Do We Still Need Old Vaccines?

**DOI:** 10.3390/vaccines10060891

**Published:** 2022-06-02

**Authors:** Maria Isaguliants, Felicity Jane Burt

**Affiliations:** 1 Institute of Microbiology and Virology, Riga Stradins University, LV-1007 Riga, Latvia; 2 N.F. Gamaleya National Research Center for Epidemiology and Microbiology, 123098 Moscow, Russia; 3 Peoples Friendship University of Russia (RUDN University), 117198 Moscow, Russia; 4 Division of Virology, National Health Laboratory Service, Universitas, Bloemfontein 9300, South Africa; 5 Division of Virology, Faculty of Health Sciences, University of the Free State, Bloemfontein 9300, South Africa

Until December 2019, we were living in the world of successfully functioning vaccines and vaccination programs. Smallpox was eradicated, polio close to being eradicated, HBV infection controlled in endemic regions, flu counteracted by seasonal vaccinations, previously deadly bacterial infections almost forgotten; respective diseases often not recognized by the medical doctors when occasional cases occurred [1]. A breakthrough finally came for malaria, promising an effective vaccine in a relatively near future [2]. Absence of success with the HCV vaccine was almost forgotten due to the success of directly acting antiviral drugs [3]. This gradual successful progress in vaccine development was marred by an inability to create an efficacious HIV-1 vaccine despite years of hard work and billion dollar/EUR investments, but, again, as for HCV, therapy with highly active antiretroviral drugs turned the infection into controllable chronic disease, with people living with HIV-1 having nearly the same life expectancy and quality of life as non-infected individuals [4,5]. Problems remained with the costs of vaccines and their availability to low-income countries, mass migration requiring re-vaccinations and re-introduction of vaccines into the areas earlier considered to be non-endemic for the disease, as well as vaccine hesitancy, with which society coped with a moderate success. In this idyllic scenario, we considered the gradual replacement of preclinical vaccine trials in experimental animals with in vitro models [6] and practiced a very careful late-phase introduction of new vaccines into clinical trials to ensure 100% safety. Our conservatism and cautiousness hindered clinical applications on the new vaccines based on the “naked” nucleic acids, RNA as well as DNA, recognized as promising more than 20 years ago, but not implemented until by 2020 [7]. This panorama of conservative vaccine development was blown up by the severe acute respiratory syndrome coronavirus 2 (SARS-CoV-2) pandemic.

SARS-CoV-2 pandemic has highlighted the importance of vaccine development and the role of vaccines in the disease prevention. It also put on trial the position of the traditional vaccine approaches based on the inactivated or live-attenuated microbial vaccines. Historically, the most successful vaccines of the pre-COVID-19 past have been based on the attenuated live viruses. To date, the yellow fever vaccine remains one of the most efficacious vaccines against an infectious pathogen. However, attenuated vaccines have distinct disadvantages, such as the risk to revert to the virulent wild type, as well as limitations associated with administration of these vaccines to immunocompromised individuals. Inactivated vaccines provided a safer option, however they are known to be less immunogenic than the live attenuated vaccines, which demands more frequent booster vaccinations. Additionally, they primarily induce humoral immunity with limited T-cell responses, thus limiting application against pathogens for which protection is based on cellular immunity.

Thanks to the lessons learnt from the COVID-19 pandemics, these traditional vaccines and vaccination approaches may now be gradually, possibly rapidly, replaced with the novel ones exploiting modern technologies, such as viral vectors, synthetic nucleic acid (both RNA and DNA), virus-like particles, nanoparticles, and recombinant, often chimeric, protein subunits expressed from the synthetic genes. We have also matured to understanding of the necessity of adequate animal disease/infection models enabling tests of the efficacy of antimicrobial drugs and vaccines [8], alongside with the principle preparedness to perform pathogen-challenge tests directly in humans [9].

Nowadays, two years after the start of the COVID-19 pandemic, we evidence the amazing development in the field of vaccinology towards new vaccine platforms, proven to be safe after the wide application of COVID-19 vaccines. DNA vaccines have previously shown promising results in animal models, but failed to induce comparably strong responses in non-human primates. Recent strides in technological advancements, particularly, with regard to the delivery systems and adjuvants applied to SARS-CoV-2 DNA vaccine development, placed DNA vaccines in a favorable position to induce efficacious responses [10,11]. Hundreds of scientists had worked on mRNA vaccines for decades before, but the breakthrough came only with SARS-CoV-2 pandemic which mRNA vaccines were able to actually limit [12]. This turned mRNA vaccines given to hundreds of millions of people around the world into the most important and profitable ones in the history of mankind with global sales in 2021 topping USD 50 billion [13]. The other new and, by now, widely applied option is viral vector-based vaccines. The advantage of using viral vector-based delivery systems over the ”naked” nucleic acid vaccines is that viral vectors and replicon-based vectors can infect cells. The replication process mimics natural infection, similar to live attenuated vaccines, stimulating both humoral and cellular responses. Antigen delivery systems have been investigated using viral vectors, including DNA vectors, such as adenoviruses and poxviruses, as well as RNA viral vectors, such as alphaviruses and flaviviruses. Replicating poxviruses and adenoviruses have been shown to induce good immune responses, however they have a disadvantage: they are considered as genetically modified organisms capable of replicating, with significant forthcoming biosafety issues. Non-replicating or single (one-round/abortive) replication systems are preferable, since they exclude the risks of reversion to virulence. Viral-vectored vaccines for human pathogens have been in development for decades prior to the current pandemic. This background of development was extremely helpful and highly beneficial; it provided platforms first for the current Ebola virus vaccine [14] and then to SARS-CoV-2 vaccines [15]. Progress has also been achieved in the development of veterinary vaccines using replication-deficient vectors derived from various animal adenoviruses [16]. The nucleic acid-based vaccine technology will play an increasingly important role in the future, its broad introduction facilitated by the critical positive impact of the novel nucleic acid-based vaccines on the development of SARS-CoV-2, alongside the availability of extensive safety data, ability to scale-up production, and the adaptability of these platforms to the emerging threats from the infectious pathogens as well as to cancer therapy.

These multiple-vaccine platforms and approaches are required to address the challenges posed by the emerging pathogens and provide the breadth of immunity required to withstand them. A critical role in this development will be played by adjuvants with research focused on the new ways to incorporate adjuvants into vaccine formulations to facilitate therapeutic vaccine applications. Previously important for the inactivated vaccines, they are nowadays applied for the enhancement of responses induced by the subunit vaccines since both, by the nature of their construction, are less immunogenic than replicating vaccines. An attractive alternative to such inactivated and subunit vaccines is offered by virus-like particles (VLPs), which have both a strong safety profile and less dependence on the adjuvants for immunogenic performance. The availability of human vaccines against hepatitis B and human papillomaviruses (HPV) based on VLP technology pays testimony to the effectiveness of this approach; multiple VLP-based vaccines are already in clinical trials for the renown “classical” public health threats, such as malaria and tuberculosis, as well as the recently emerged Zika virus disease [17].

This development raises important questions. Would old vaccines be gradually exchanged for new-generation vaccines? Should we purposefully replace our “old/pre-COVID-19 world” vaccines, adjuvants, and delivery technologies with the new ones, is it worth and will it pay back to perform such an exchange? Or should we limit the application of new-generation vaccines to the areas where we do not yet have good vaccine coverage? The collection of research articles selected for inclusion into this special edition provides a tentative answer to this question. The selection focuses on understanding the immunogenicity of new-generation vaccines against “old/pre-COVID-19 world” pathogens of public health concern, on novel adjuvants to improve the efficacy of the new as well as traditional vaccines, and immunomodulation required for the successful therapy of chronic viral infections and cancer, as well as strategies for the monitoring of vaccination status and vaccine efficacy, i.e., on the ways to apply the vaccine knowledge acquired during pandemics to the creation of new as well as improvement of the old/traditional vaccines.

In the stream of studies covering novel DNA and RNA-based vaccine candidates, Tipih et al. described the immunogenicity of a DNA-based Sindbis replicon expressing the nucleoprotein (NP) of the Crimean–Congo hemorrhagic fever virus (CCHFV) using a mouse model [18]. CCHFV is listed as a priority pathogen by the World Health Organization due to the lack of specific treatment or prophylactic vaccine. A replicon based on the Sindbis virus-vector encoding the complete open reading frame of a CCHFV NP was constructed and the expression of the NP characterized using transfected human embryonic kidney cells prior to immunization studies in a mouse model. The vaccine was able to induce a detectable antibody response in mice alongside with increased levels of interferon gamma and interleukin-2 from activated mouse splenocytes. Based on the cytokine spectrum and antibody profile, this candidate replicon vaccine was concluded to induce a predominantly Th1-type response.

A modified mRNA approach was described by Starostina et al. in their paper entitled “Construction and immunogenicity of modified mRNA-vaccine variants encoding influenza virus antigens” [19]. To compare head-to-head the various protocols of the design of highly immunogenic mRNA vaccines, they constructed eight mRNA variants encoding a green fluorescent protein with different modifications. The constructs that provided the most intensive fluorescence of transfected cells, i.e., the highest level of expression of encoded proteins, were then used for template synthesis from mRNA-encoded influenza immunogens. An animal model was used to identify protocols for designing mRNA vaccines that were highly immunogenic, but had a low toxicity.

In the stream of studies covering vaccine candidates based on the microbial vectors, Sergeeva et al. described the outcome for the trials of a mucosal influenza vector vaccine expressing *M. tuberculosis* antigens TB10.4 and HspX antigens in mice and guinea pigs [20]. Tuberculosis remains a significant public health disease in many parts of the world. A mucosal approach appeared to be preferable to systemic immunization due to the airborne nature of the infection. In a mouse model, the candidate vaccine provided comparable protection against two virulent *Mycobacterium tuberculosis* strains to subcutaneous BCG immunization. In a guinea pig model, the candidate vaccine boosted the response induced in a heterologous prime-boost formulation using BCG prime immunization, and improved protection against *M tuberculosis*.

Despite the express wide introduction of the novel vaccination technologies, an interest pertains in further development of the traditional vaccines and vaccination approaches. A good example is the inactivated polio vaccine that is of high significance for public health. An increase in the global availability of the polio vaccine is high public-health demand. The inactivated polio vaccines are in specific demand, since the existing oral attenuated vaccine can evolve into virulent forms and cause outbreaks of the disease [21]. To ensure the provision of inactivated polio vaccines, Pinieava et al. performed a study “Immunogenicity and safety of inactivated Sabin-strain polio vaccine “PoliovacSin”: clinical trials phase 1 and 11” describing clinical trials of the inactivated polio virus vaccine [22]. The inactivated vaccine PoliovacSin was prepared based on the live attenuated Sabin strain. A randomized, double-blind placebo-controlled clinical trial included 60 participants who received one dose of PoliovacSin or Placebo and a phase-II multicenter, randomized, double-blind, comparative clinical trial involving 200 participants who received one dose of PoliovacSin or Imovax Polio. Overall, the inactivated vaccine was well tolerated, had a good safety profile that induced high-neutralizing antibody levels to polioviruses types 1–3 (Sabin and wild virus). Clinical trials confirmed good tolerability, low reactogenicity, and a high safety profile of the PoliovacSin and pronounced immunogenic properties, offering this vaccine for wide application in the community vaccination programs.

As mentioned in our introduction, adjuvants can assist by significantly enhancing the immune responses. Chen et al. described the effectiveness of the alum Pickering emulsion as an adjuvant to improve the efficacy of the candidate malaria vaccine [23]. Malaria remains an important public health concern with significant fatalities recorded annually. As stated by the authors, potential vaccines ideally require antigens from each stage of the parasite life cycle or epitopes of multiple key antigens of the Plasmodium parasite. Adjuvants can enhance responses, however the safety of the adjuvants as well as their capacity to enhance the immunogenicity must be evaluated prior to systematic trials of vaccine candidates. The authors used aluminum hydroxide gel (“alum”) as a stabilizer to prepare alum-stabilized Pickering emulsions (ALPE), including variants containing an immunostimulant monophosphoryl lipid A (ALMPE), incorporated in order to enhance the immune response. In vitro studies suggested a higher antigen load could be reached in antigen presenting cells using the adjuvant and immunostimulant compared to alum. ALMPE was used as an adjuvant to enhance the immunogenicity of the control immunogen ovalbumin, and of the prototype malaria vaccine - multi-epitope chimeric antigen containing a selection of malaria epitopes. In mice, use of ALMPE allowed to obtain antibody responses comparable to those obtained using Freund’s adjuvant (FA), but without side effects usually observed when immunogens are administered with FA [23].

Adjuvant effects are crucial not only for the development of efficient vaccines against infectious diseases, but also, and even more so, in the immunotherapy of cancer where efforts are needed to overcome cancer-induced immune modulation and immune suppression [24]. In a murine model of human breast cancer, Trofimova et al. described the anti-tumor immunomodulatory effects of interferon gamma delivered by alphaviral vector [25]. Previous preclinical and clinical studies of the anti-tumor immunomodulatory properties of IFN-γ produced inconsistent results necessitating the need for further investigations. The authors developed a replication-deficient Semliki Forest virus vector expressing interferon gamma (SFV/IFNg) and described the anti-tumor effects when using it against 3D spheroids in vitro and in a syngenic mouse model in vivo. Spheroid growth was inhibited in the presence of the expressed IFNg. In vivo, SFV-driven interferon IFNg expression was shown to inhibit growth and metastatic activity in mice of murine mammary gland adenocarcinoma 4T1 cells, a powerful murine model of human breast cancer. The SFV/IFNg vector was also shown to induce a therapeutic anti-tumor T-cell response [25].

One of the fundamental requirements for the development of next-generation vaccines is the understanding or adequate presumption of the immune correlates of the protection and identification of the appropriate components of a pathogen to be incorporated into the vaccine to induce protective immunity. Not least important is the assessment of the vaccine efficacy with regard to what parameters to monitor. There are challenges in the identification of the predictive markers of immunogenicity that align with immune correlates of protection. The collection of papers in this Special Issue included two review articles devoted to monitoring vaccine efficacy. In a review entitled “Predictive markers of immunogenicity and efficacy for human vaccines”, van Tilbeurgh et al. provided an overview of the vaccine-immune signatures in preclinical models and in the target human populations [26]. The authors discussed high-throughput technologies for probing vaccine-induced responses and analyses of data and predictive modeling to determine vaccine efficacy. In the second, not least comprehensive, review entitled “Strategies for immunomonitoring after vaccination and during infection”, Adam et al. used the recent pandemic to provide examples of approaches to immunomonitoring that could be applied for assessing vaccine efficacy [27]. The parameters described by these two reviews are important for the selection of vaccine candidates for large-scale preclinical development and clinical testing. The next step is to monitor the vaccine efficacy in the clinical application, constituting an important part of vaccination programs. Mikhailov et al. presented an article entitled “Universal single-dose vaccination against hepatitis A in children in a region of high endemicity” [28]. The authors performed a cross-sectional study to assess the immunological and epidemiological effectiveness of hepatitis A vaccination programs over a five-year period after the commencement of the program. The Tuva region had previously recorded high anti-HAV antibody-detection rates in children pre-vaccination (66%). The vaccine program reduced the incidence of infection in children from 450–860 per 100,000 to 7.5 per 100,000 in children below 18 years old, and to 3.2 per 100,000 in the total population one year after introduction. The data vividly proves that a single-dose vaccination program is effective in the control of hepatitis A, even in the high-endemicity regions [28].

Vaccine development plays an important role in managing communicable and non-communicable diseases. New vaccines and new vaccination approaches will gradually change the vaccines we are currently using, improving some and replacing others. The successful development and use of all, new as well as “old”, vaccines require an understanding of the immune correlates of protection, application of multiple platforms to address the diversity of pathogens and cancers, development of new adjuvants to improve vaccine effectiveness as well as better strategies to monitor the immunogenicity, efficacy and reduction in disease burden from the application of the new and updated traditional vaccination programs.
